# Impact of chemotherapy on humoral and cellular immune responses to COVID-19 vaccination in patients with solid tumors

**DOI:** 10.3389/fimmu.2025.1664072

**Published:** 2025-09-25

**Authors:** Andrea Favalli, Giorgio Patelli, Paola Gruarin, Andrea Gobbini, Elisa Pesce, Sara Mariano, Mauro Bombaci, Francesca Vincenti, Lorena Donnici, Silvia Marchese, Daniele Piscazzi, Alessio Amatu, Federica Tosi, Silvia Ghezzi, Arianna Pani, Silvia Principato, Andrea Lombardi, Alessandra Bandera, Sergio Abrignani, Salvatore Siena, Andrea Sartore-Bianchi, Renata Grifantini

**Affiliations:** ^1^ INGM, Istituto Nazionale Genetica Molecolare “Romeo ed Enrica Invernizzi”, Milan, Italy; ^2^ Department of Oncology and Hemato-Oncology, Università degli Studi di Milano, Milan, Italy; ^3^ ⁠Niguarda Cancer Center, Grande Ospedale Metropolitano Niguarda, Milan, Italy; ^4^ Department of Clinical Sciences and Community Health, University of Milan, Milan, Italy; ^5^ DiSFeB Dipartimento di scienze farmacologiche e biomolecolari, Università degli studi di Milano, Milan, Italy; ^6^ Infectious Diseases Unit, Foundation IRCCS Ca’ Granda Ospedale Maggiore Policlinico, Milan, Italy; ^7^ Department of Pathophysiology and Transplantation, University of Milan, Milan, Italy; ^8^ CheckmAb Srl, Milan, Italy

**Keywords:** solid tumors, SARS-CoV-2, vaccinations, seroconversion, memory B cells, high-dimensional, unbiased immunophenotyping

## Abstract

**Introduction:**

Despite SARS-CoV-2 pandemic has subsided, vaccine response profiling in patients with cancer remains critical.

**Methods:**

We longitudinally assessed humoral and cellular immunity in adults with solid tumours treated with chemotherapy (ChT) or non-ChT regimens after two mRNA vaccine doses plus booster, compared with vaccinated cancer-free controls, naturally infected (convalescent) subjects including both patients with cancer and cancer-free individuals, and unvaccinated/uninfected individuals with or without cancer as a baseline reference.

**Results:**

Anti-Spike IgG titres matched cancer-free controls, but anti-RBD titres and neutralising activity were consistently lower in cancer post-vaccination, most markedly with ChT, and declined faster over 4-6 months. Boosters restored IgG, yet gains were smaller in ChT recipients. Cellular analyses revealed sustained and booster-enhanced Spike-specific B cells in all groups; however, ChT exposure was associated with reduced CD27 expression on these cells, suggesting impaired activation and memory maturation.

**Discussion:**

These findings support tailored immune monitoring and vaccination strategies in oncology and identify CD27 downregulation as a novel B-cell dysfunction detected by high-dimensional immunophenotyping.

## 1 Introduction

The coronavirus disease 2019 (COVID-19) has posed a significant threat to global public health. Originating in the city of Wuhan, China, in late 2019, the severe acute respiratory syndrome coronavirus 2 (SARS-CoV-2) spread rapidly and has since caused more than 7 million confirmed fatalities worldwide, making it one of the most lethal pandemics in contemporary history (1). The adoption of non-pharmaceutical interventions to limit contagion, followed by the subsequent initiation of a persuasive international vaccination campaign in 2021, has mitigated some of the devastating consequences. However, as of January 2025, more than 500 deaths per week have been still documented according to the World Health Organization (WHO- https://data.who.int/dashboards/covid19).

Oncological status has been an important risk factor for COVID-19. Individuals with cancer are more susceptible to infections due to coexisting chronic disease, poor overall health status, and systemin immunosuppression (2, 3). These patients were found to have a substantial risk of developing severe COVID-19 and dying from the disease (3, 4). Additionally, cancer intensifies chronic inflammation, favoring the release of inflammatory cytokines that contribute to COVID-19 clinical features (5).

Increased risk has been highlighted for patients harboring hematological tumors, respiratory tract cancers, or those receiving cytotoxic drugs (3, 4). Additionally, several interplay mechanisms between COVID-19 and cancer were proposed, including CD4^+^FOXP3^+^ regulatory T cell (Treg) enrichment, T cell lymphopenia, T-cell exhaustion related to tumor immune-escape, and myelotoxicity from active anti-cancer treatments (6–8). However, scientific evidence thus far has been inconclusive when focusing on patients with solid tumors. In particular, while an impaired humoral response, exhausted T cell phenotype and prolonged viral shedding have been observed in most patients with leukemia and lymphomas, a more subtle impact on the immune system seems to characterize virus-exposed patients with solid tumors, who generally develop immune signatures resembling those of COVID-19 patients without cancer (9, 10).

Additionally, patients with cancer were excluded from initial COVID-19 vaccine trials, leaving them unaddressed during the development of novel mRNA vaccines (BNT162b2 and mRNA-1273) and adenovirus-vectored vaccines (ChAdOx1 nCoV19, Ad26.COV2-S and Gam-COVID-Vac) (11). Subsequent independent studies have documented poorer immunogenic response to vaccination in this population compared to cancer-free controls, both in terms of anti-spike (S) antibodies and neutralizing activity of the receptor-binding domain-angiotensin-converting enzyme 2 (RBD-ACE2) (11). Overall, vaccination was found to be safe and nevertheless effective, although seroconversion rates and the magnitude and duration of immune response were lower than those observed in cancer-free controls (11–13). This effect was particularly evident in, but not limited to, patients with hematological malignancies. We and others have previously reported that up to 6% of individuals with solid tumors receiving anti-cancer therapy do not develop seroconversion after primary (two-dose) mRNA vaccination, compared to 0.2% in controls (11, 13, 14). Although booster dose strategies improved seroconversion rates, the estimated risk of persistent seronegativity remains around 30% according to our and other data (13, 15–17). Cancer is considered an independent risk factor for poor vaccine immune response (11, 18). Across different studies, inconsistent results have emerged regarding the impact of cytotoxic chemotherapy (ChT) and chronic steroid administration as putative negative factors for seroconversion (11, 18) We have previously reported that, even though our study was not powered to detect an association between seroconversion and different types of anticancer agents, the vast majority (80%) of patients who did not achieve seroconversion were receiving cytotoxic agents (14, 16). In addition, we found that poor clinical condition (defined as an ECOG performance status >2) was the main factor exerting a significant negative impact on seroconversion, a finding that has been corroborated by others (14, 19–21).

Understanding the immunological aspects of SARS-CoV-2 infection and vaccination in patients with solid tumors is crucial for optimizing effective prevention and vaccination strategies in the event of a COVID-19 resurgence or future pandemics. Indeed, the role of clinical variables alone in influencing immunogenicity is not yet conclusive (11). In-depth immunological studies of the humoral and cellular immune response against COVID-19 vaccine in this population are limited in number and warrant further investigation (22–24). For this reason, in this study we performed an integrated, multiparametric characterization of the humoral and cellular adaptive immune responses in patients with solid tumors undergoing active treatments, compared to cancer-free controls and unvaccinated infected cancer patients, with the specific question of how cytotoxic ChT impacts these responses compared to other anticancer treatments, including sole immunotherapy with checkpoint inhibitors.

## 2 Materials and methods

### 2.1 Study design and patient accrual

The primary objective of this prospective, single-center, multi-cohort, observational study was to characterize the humoral and cellular immune responses to COVID-19 vaccination in patients with solid tumors undergoing active treatment, with a specific focus on the effect of ChT vs non-ChT regimens. These responses were compared to those in non-vaccinated cancer and cancer-free patients with natural immunity due to SARS-CoV-2 infection, and vaccinated cancer-free individuals.

The study included adult patients (≥18 years) capable of providing written informed consent. Blood samples were collected at different time points from patients with solid tumors vaccinated with the Pfizer-BioNTech mRNA vaccine (Comirnaty, BNT162b2) who received anti-cancer treatment within 3 months prior to inclusion. As comparators, we included: i) individuals without a cancer diagnosis and not receiving immune-modulating treatments who were vaccinated for SARS-CoV-2 during the same period; ii) unvaccinated individuals, either with and without a cancer diagnosis, who experienced SARS-CoV-2 infection confirmed by nasopharyngeal swab (referred to as convalescents); iii) unvaccinated, uninfected individuals both with and without a cancer diagnosis, recruited prior to any SARS-CoV-2 exposure, and used to define pre-vaccination baseline immune responses. All patients with cancer must have received treatment within the 3 months prior to inclusion.

Blood samples were collected from vaccinated individuals at multiple time points designed to reflect key phases of vaccine-induced immunity, according to previous studies (11–14): within 2 months after the second vaccine dose (“T1” cohort), within 4–6 months after the second dose (“T4-6” cohort), and within 3 months after a third booster vaccination (“T1b” cohort). Blood samples from unvaccinated individuals recruited before any SARS-CoV-2 exposure served as baseline (“T0” cohort). Blood samples from unvaccinated convalescents were collected between 20–50 days and 2–4 months after SARS-CoV-2 diagnosis for patients with cancer and cancer-free controls, respectively. Patients with cancer were reclassified at each time point as receiving ChT or non-ChT according to the treatment administered at the time of blood sampling.

From April 2020 to January 2022, individuals who fulfilled the above-mentioned inclusion criteria were invited to participate in the study. Data from medical records were annotated through a REDCap electronic data platform (25). Patients with cancer were classified upon cytotoxic ChT exposure into ChT versus non-ChT treated, the latter receiving one of the following treatments or their combinations: tyrosine kinase inhibitors, monoclonal antibodies including immune checkpoint inhibitors, and endocrine therapies.

The study was conducted in accordance with the Declaration of Helsinki and the Good Clinical Practice guidelines. All participants provided written informed consent prior to sample collection. Protocol numbers and ethics approval a reported in the Ethics approval section.

### 2.2 Analysis of spike-specific B cell response

Spike-specific memory B cells were detected by SARS-CoV-2 spike B cell Analysis kit (Miltenyi Biotec cat. no. 130-128-022), according to manufacturer’s instructions. Briefly, recombinant SARS-CoV-2 biotinylated spike (0.1 mg/ml) was incubated with Streptavidin-PE or PE-Vio 770 for 15 minutes at room temperature. PBMC were thawed and CD19^+^ cells were enriched using the REAlease CD19 MicroBead kit (Miltenyi Biotec cat. no. 130-117-034). Enriched B cells were counted and resuspended in antibody staining mix containing anti-CD19 APC-Vio770; anti-CD27 Vio Bright FITC; anti-IgG VioBlue; anti-IgM APC; anti-CD38 BB700; anti-CD138 PECF-594; anti-CD21 PE-Cy5 and 0.15 or 0.3 µg/mL spike conjugate with PE or PE-Vio 770 (according to Miltenyi Biotec cat. no. 130-128–022 protocol). After incubation at 4 °C, cells were washed and acquired on BD FACSymphony A5 cytometer (BD Biosciences). The gating strategy for the identification of spike-specific B cells is represented in **Supplementary Figure S1**. The LOQ of spike-specific B cells was arbitrarily set to 0.0001%.

### 2.3 Unsupervised analysis of flow cytometry data

Unsupervised analysis was performed as previously described (26, 27). Immunophenotyping by high-dimensional flow cytometry is described in the **Supplementary Materials** and in **Supplementary Figures S1**–**S3**). Briefly, each compensated sample was randomly subsampled to 1038 cells and data were exported to a Flow Cytometry Standard (FCS) file. FCS files were imported into R (v4.3.2) through the read.flowSet function from the flowCore (v2.14.2) R package. We applied the Logicle transformation that allows the use of multiple samples to estimate transformation parameters. To reduce batch effect due to technical (non-biological) variation we normalized the signal of each marker with the function gaussNorm from the flowStat package (v4.14.1). Then, samples were concatenated into a SingleCellExperiment object in R using the function prepData from the CATALYST R package (v1.26.0). Environment seed was set equal to 1234. Dimensionality reduction by UMAP was subsequently applied to visualize relative proximities of cells within reduced dimensions by runDR function with 15 neighbors and excluding CD19 marker from the features. We performed high-resolution, unsupervised clustering using the Rphenograph package (v0.99.1.9003) with k parameter set to 50, finding a total of 16 clusters. Clusters were manually explored and integrated into 7 clusters resembling as many B cell subsets. To limit variability during different runs set.seed function was used with 1234 as unique parameter.

### 2.4 Statistical analysis

Statistical analysis was performed using GraphPad (Prism 10.2.3 software). Descriptive statistics were calculated to summarize baseline characteristics, presented as medians with interquartile ranges (IQRs) for continuous variables and frequencies with percentages for categorical variables. According to normality continuous data were analysed using Kruskal-Wallis test followed by Multiple Mann-Whitney test for non-parametric distribution, while parametric distribution were compared by Mixed-effect model with Geisser-Greenhouse correction followed Tukey’s test for multicomparisons. Dunn’s correction was used for multiple comparisons.

## 3 Results

### 3.1 Patient characteristics

From April 2020 to January 2022, 82 patients with cancer were enrolled. Blood samples were collected from 9 patients before any exposure to SARS-CoV-2 or vaccination (T0), 29 patients with confirmed SARS-CoV-2 infection (convalescents), and 41 patients who received the COVID-19 mRNA vaccine BNT162b2. Patient characteristics are presented in **Table 1A**. For a subset of individuals, samples were collected at multiple time points to capture the dynamics of the immune response over time. Post-vaccination samples were taken at different time points: within one-two months after primary vaccination consisting of two mRNA vaccine doses (T1), six months after primary vaccination (T4-6), and one month after the boosting dose (T1b). The median time between anti-cancer treatment administration and blood sampling for analyses was 15 days (IQR 0-27), indicating that most samples were collected during active treatment. An interval of 0 days indicates blood withdrawal immediately before a new treatment cycle. As cancer-free controls, we included a cohort of cancer-free vaccinated subjects not undergoing immune-suppressive treatments (N = 27, **Table 1B**), who had received SARS-CoV-2 vaccination during the same period and whose samples were collected at similar timepoints; 12 of these had also experienced SARS-CoV-2 infection before vaccination.

**Table 1:** Demographic and clinical characteristics of patients with cancer and cancer-free individuals.

A. Characteristics of patients with cancer by type of cancer treatment.			
	Patients with cancer		
	All	ChT	non-ChT
Number of patients (%)	82	46 (56.1)	36 (43.9)
Age at enrollment (median [IQR])	68 [58-73]	69 [60-75]	66 [55.5-73]
Body mass index (median [IQR])	24 [23-28]	24 [22-26]	23 [23-27]
Sex (%)			
Male	41 (50)	21 (45.7)	20 (55.6)
Female	41 (50)	25 (54.3)	16 (44.4)
ECOG performance status (%)			
0	24 (29.3)	10 (21.7)	14 (38.9)
1	38 (46.3)	23 (50.0)	15 (41.7)
≥2	20 (24.4)	13 (28.3)	7 (19.4)
Smoking status (%)			
Never	27 (32.9)	15 (32.6)	12 (33.3)
Former or current	41 (50.0)	23 (50.0)	18 (50)
NA	14 (17.1)	8 (17.4)	6 (16.7)
Comorbidities (%)			
At least one comorbidity	62 (75.6)	32 (69.6)	12 (33.3)
No	19 (23.2)	13 (28.2)	24 (66.7)
NA	1 (1.2)	1 (2.2)	0 (0.0)
Primary tumor type (%)			
Thoracic	26 (31.7)	12 (26.1)	14 (38.9)
Gastrointestinal	29 (35.4)	20 (43.5)	9 (25.0)
Genitourinary, breast and others	27 (32.9)	14 (30.4)	13 (36.1)
Tumor stage (%)			
Locally advanced	9 (11.0)	9 (19.6)	0 (0.0)
Metastatic or unresectable	73 (89.0)	37 (80.4)	36 (100.0)
Steroids			
Yes (any dose)	27 (33.0)	15 (32.6)	12 (33.3)
No	54 (65.8)	30 (65.2)	24 (66.7)
NA	1 (1.2)	1 (2.2)	0 (0.0)
B. Characteristics of non-cancer individuals			
Number of individuals		27	
Age at enrollment (median [IQR])		53 [42-61]	
Sex (%)			
Male		14 (51.8)	
Female		13 (48.2)	

ChT, Cytotoxic chemotherapy; non-ChT, Non-cytotoxic chemotherapy.

As a comparative benchmark for the immune response elicited by natural SARS-CoV-2 infection, we included 32 patients with cancer and 11 cancer-free subjects referred to as convalescents, who provided blood samples at 20–50 days and 2–4 months from a positive swab, respectively.

Patients with cancer received various types of anticancer systemic treatments during the *continuum* of care. We grouped patients according to the type of treatment they were receiving at the time of vaccination, with 46 patients receiving ChT and 36 receiving chemo-free regimens, i.e., targeted agents including but not limited to immunotherapy based on checkpoint inhibitors (non-ChT), and hormone therapy. No difference was found between ChT and non-ChT groups regarding the main demographic and clinical characteristics.

Humoral and cellular immune responses were assessed in these samples as schematized in **Supplementary Figure S4**.

### 3.2 Dynamics of SARS-CoV-2 anti-spike and anti-RBD IgG response to BNT162b2 vaccine in patients with cancer and influence of the cancer treatment

We evaluated the antibody response induced by the BNT162b2 vaccine over time by ELISA, measuring serum IgG titers against the recombinant spike and receptor-binding domain (RBD) of SARS-CoV-2.

**Figure 1:**
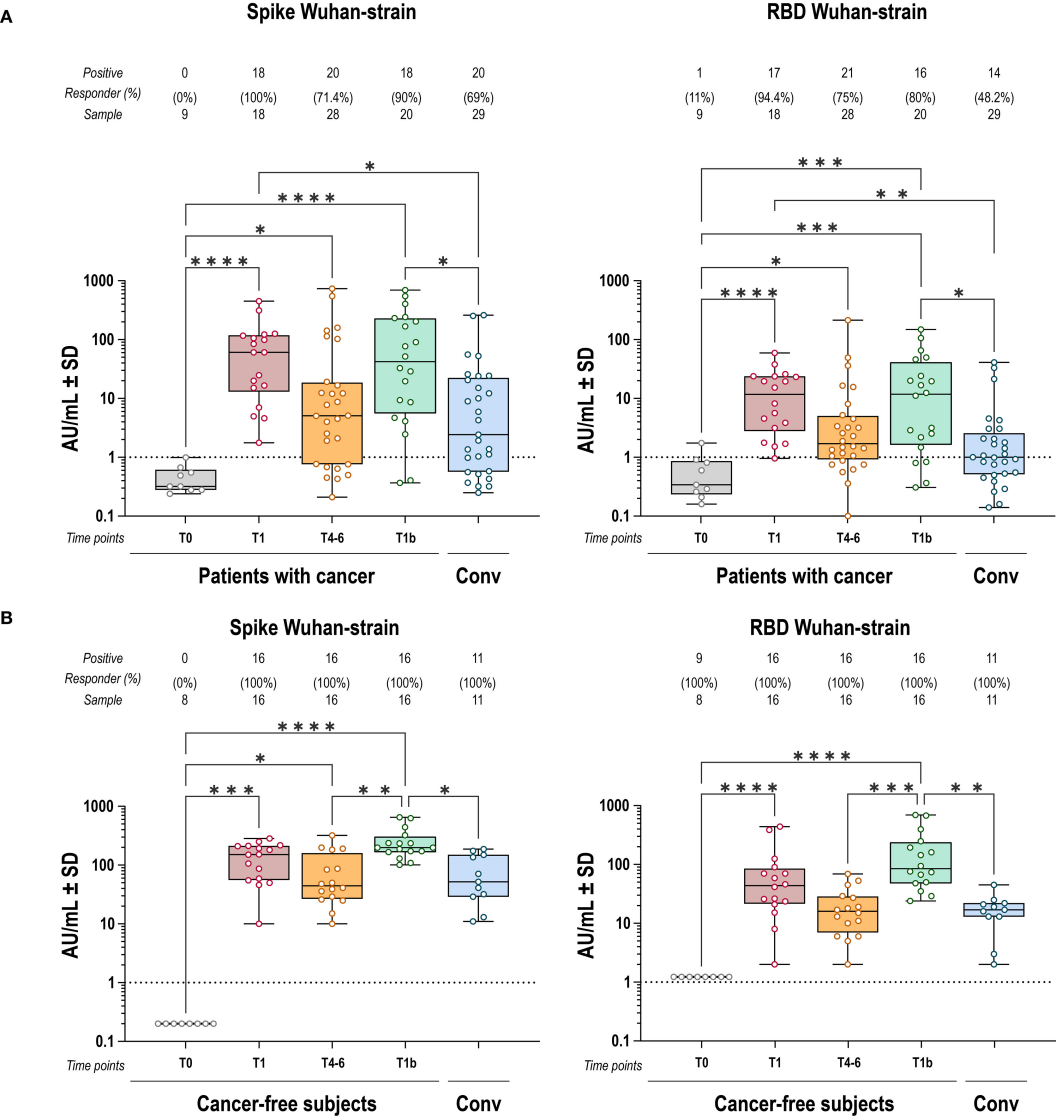
IgG response to spike and RBD Wuhan strain induced by BNT162b2 vaccine. Anti-S (left) and anti-RBD (right) IgG antibody responses to Wuhan-strain SARS-CoV-2 after COVID-19 vaccination, in cancer **(A)** and cancer-free controls **(B)**. Each dot represents a single individual, dotted lines indicate the limit of quantification (LOQ). Kruskal-Wallis test was used to compare pre- and post-vaccination samples at baseline (T0), one-two months after primary vaccination (T1), four-six months after primary vaccination (T4-6), and 1 month after the booster dose (T1b). Dunn’s correction was used for multiple comparison testing. Significance levels: ****p value <.0001, ***p value <.001, **p value <.01 and *p value <.05. Absence of significance symbols indicates that comparisons did not reach statistical significance.

**Figure 2:**
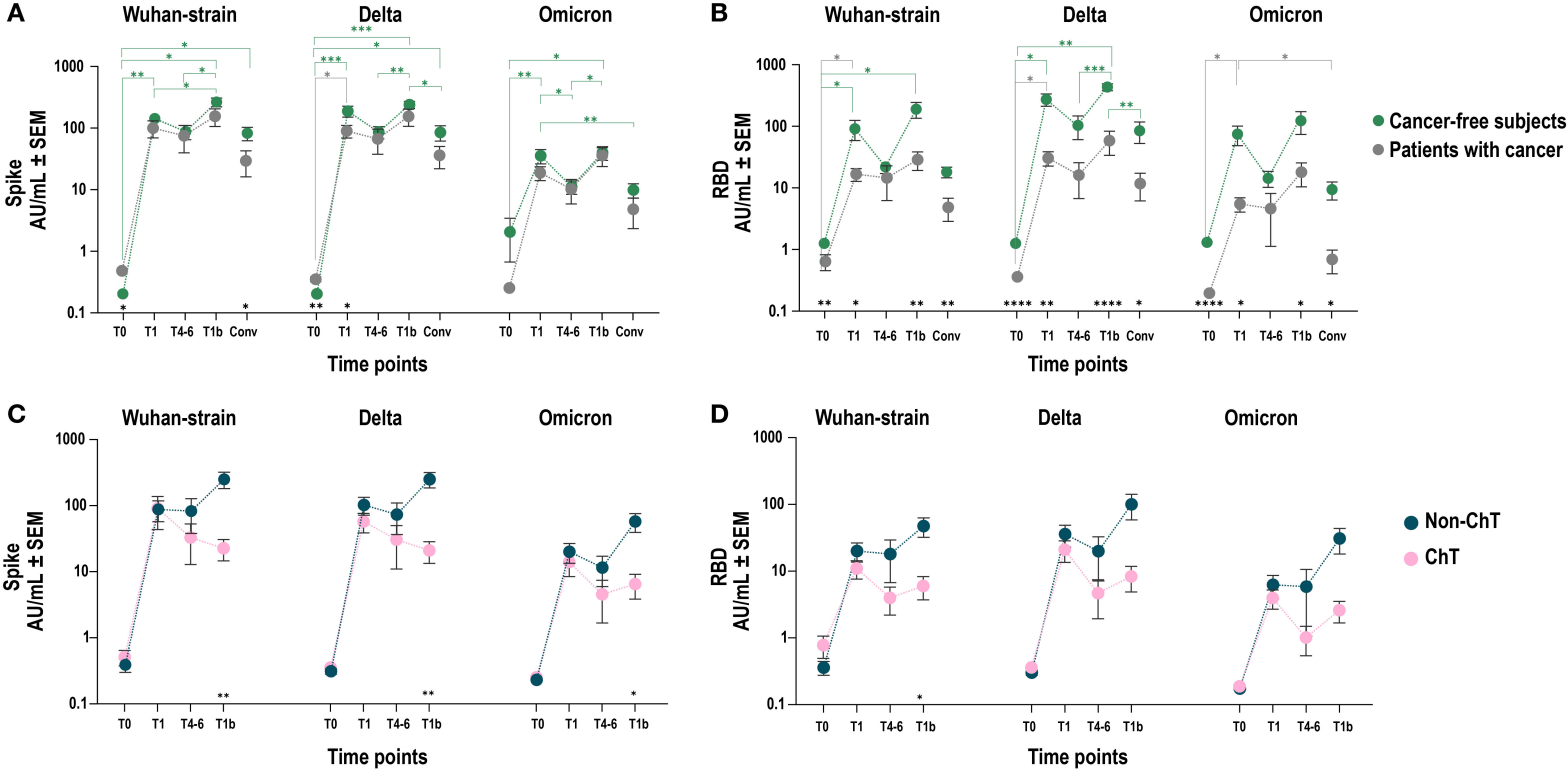
Cross-reactivity of vaccine induced IgG on spike and RBD Wuhan and corresponding VoC. Comparison of SARS-CoV-2 anti-spike **(A–C)** and anti-RBD **(B–D)** IgG antibody responses between patients with cancer (grey line and dots) and cancer-free controls **(A, B)** (grey and green line and dots, respectively) after COVID-19 vaccination in Wuhan, Delta and Omicron VoCs. Each dot represents mean values with SEM values of individual IgG titers. Mixed-effect model was fitted to the data and Fisher test was used to compare the cohorts pre- and post-vaccination at baseline (T0), one-two months after primary vaccination (T1), four-six months after primary vaccination (T4-6), and 1 month after the booster dose (T1b). Tukey’s corrections were used for multiple comparison testing. Significance levels are as follows: ****p value <.0001, ***p value <.001, **p value <.01 and *p value <.05; grey asterisks indicate statistical difference between time points within patients with cancer group; green asterisks indicate statistical difference between time points within cancer-free controls’ group; black asterisks indicate statistical difference between groups. Absence of significance symbols indicates that comparisons did not reach statistical significance.

Vaccinated patients with cancer elicited anti-S and anti-RBD IgG responses with increased levels in all individuals compared to baseline (T0). Subsequently, both decreased by T4–6 and were boosted again after the third dose at T1b (**Figure 1A**). The anti-S and anti-RBD IgG titers induced by vaccination were higher than those measured in convalescents by natural infection, both after primary vaccination (T1) and boosting (T1b). Despite similar overall dynamics to cancer-free subjects, IgG titers in patients with cancer were lower and showed greater inter-individual variability (**Figure 1B**). In particular, while comparable anti-S antibody levels were observed between cancer patients and cancer-free controls, anti-RBD Wuhan-strain IgG titers were significantly lower in patients with cancer at multiple time points (**Figure 2**).

To assess potential cross-protection against variants of concern (VoC), we measured the ability of IgG to recognize the Delta and Omicron spike and RBD in vaccinated patients with cancer and cancer-free controls. Overall, we observed comparable levels of anti-spike IgG, which recognized the Wuhan and Delta strains with higher efficacy than the Omicron (**Figure 2A**). Differently, we confirmed a lower level of anti-RBD Wuhan-strain IgG antibodies in patients with cancer, irrespective of the VoC examined (**Figure 2B**).

We next hypothesized that the generation of a productive antibody response to SARS-CoV-2 vaccination in patients with cancer could vary upon the influence of different treatment types, depending on their mechanism of action. Given the major immunosuppressive nature of ChT compared to other anticancer agents, we focused on comparing patients receiving ChT vs non-ChT agents (**Figures 2C, D**). We found that, after primary vaccination (T1), ChT and non-ChT patients elicited comparable IgG levels against spike and RBD. However, in non-ChT treated patients these levels were sustained throughout the entire vaccination period (T4-6) and further increased after the booster dose (T1b). Conversely, in ChT-treated patients the antibody response showed a trend towards shorter persistence and a statistically significantly lower responsiveness to the booster dose for both anti-spike (p < 0.01) and anti-RBD IgG (p < 0.05). Collectively, patients treated with ChT mounted a less robust response compared to non-ChT, which was also evident in the IgG recognition of the spike and RBD of the Delta and Omicron VoCs (**Figures 2C, D**).

### 3.3 Neutralizing antibody response against SARS-CoV-2 spike Wuhan and delta VoC

**Figure 3:**
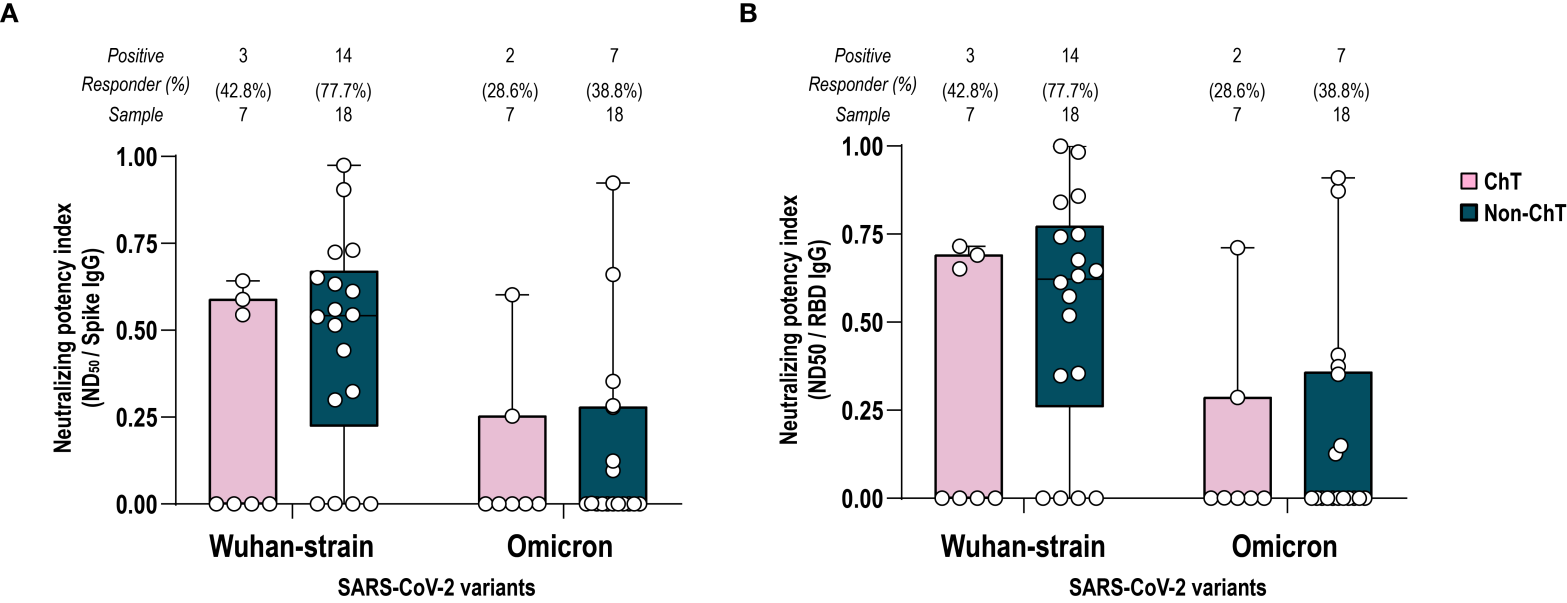
Potency index against spike Wuhan and Omicron VoC. Comparison between ChT (pink box) and non-ChT patients (blue box). Serum-neutralizing potency index was calculated as the ratio of neutralizing titer for Wuhan and Omicron VoC to spike-specific IgG titers **(A)** or to RBD-specific IgG titers **(B)**. The number and the frequency of neutralizing sera on the total sample are reported above the graphs. Kruskal-Wallis test were used to compare samples (ChT and non-ChT groups) and were corrected using Dunn’s tests. Each dot represents a single individual. Significance levels are as follows: *p value <.05. Absence of significance symbols indicates that comparisons did not reach statistical significance.

Neutralizing antibodies are an acknowledged correlate of protection against SARS-CoV-2 infection, with their dynamics and persistence over time generally reflecting the levels of anti-S and anti-RBD IgG. Given that differences in antibody persistence between ChT- and non-ChT-treated patients started to emerge at T4-6, we selected this timepoint to evaluate whether reduced binding antibody levels in the ChT group were accompanied by impaired neutralizing capacity and altered functional potency. Therefore, we assessed the ability of sera collected at T4-6 (four-six months after primary vaccination) to interfere with the Wuhan SARS-CoV-2 pseudo-viral infection of HEK293TN expressing hACE2 receptor. In addition, we evaluated the potential cross protection against omicron VoC, comparing spike-Wuhan with spike-Omicron pseudoviruses. To estimate the neutralization potency of elicited antibodies against spike and RBD, we measured the potency index, expressed as the ratio between neutralization titers to the spike and RBD specific antibody binding titers. As a trend (p > 0.05), non-ChT patients had a higher frequency of positive sera than the ChT ones. A lower potency index and positivity was observed against the Omicron VoC pseudotype, with no differences between the two patient groups, suggesting that the decline of the antibody titers at T4–6 and the lower IgG recognition of Omicron VoC reflected in a lower antibody neutralization ability (**Figures 3A, B**). The lack of detectable neutralizing antibodies in a subset of patients was not significantly associated with Performance Status or comorbidities (all p > 0.05, Fisher’s exact tests).

In general, the potency indices versus spike and RBD were comparable, in line with the knowledge that RBD is the main target of neutralizing antibodies.

### 3.4 Immunophenotype and spike-specificity of T cells induced by SARS-CoV-2 vaccination

We then investigated whether different anticancer treatments affected the immunological features of circulating CD4^+^ and CD8^+^ T lymphocytes and the elicitation of spike-specific T cells in vaccinated patients with cancer, as compared to cancer-free controls.

To characterize specific immunological features induced by vaccination, we first conducted multiparametric flow cytometry analyses of circulating CD4^+^ and CD8^+^ T lymphocytes collected at all time points (T0, T1, T4-6, and T1b), including ChT and non-ChT patients as compared to cancer-free subjects.

Overall, the immunophenotypic analysis of CD4^+^ and CD8^+^ T subpopulations revealed individual fluctuations and moderate changes over time and between treatment groups, without consistent group-level trends (**Supplementary Figures S5**, **S6**).

**Figure 4:**
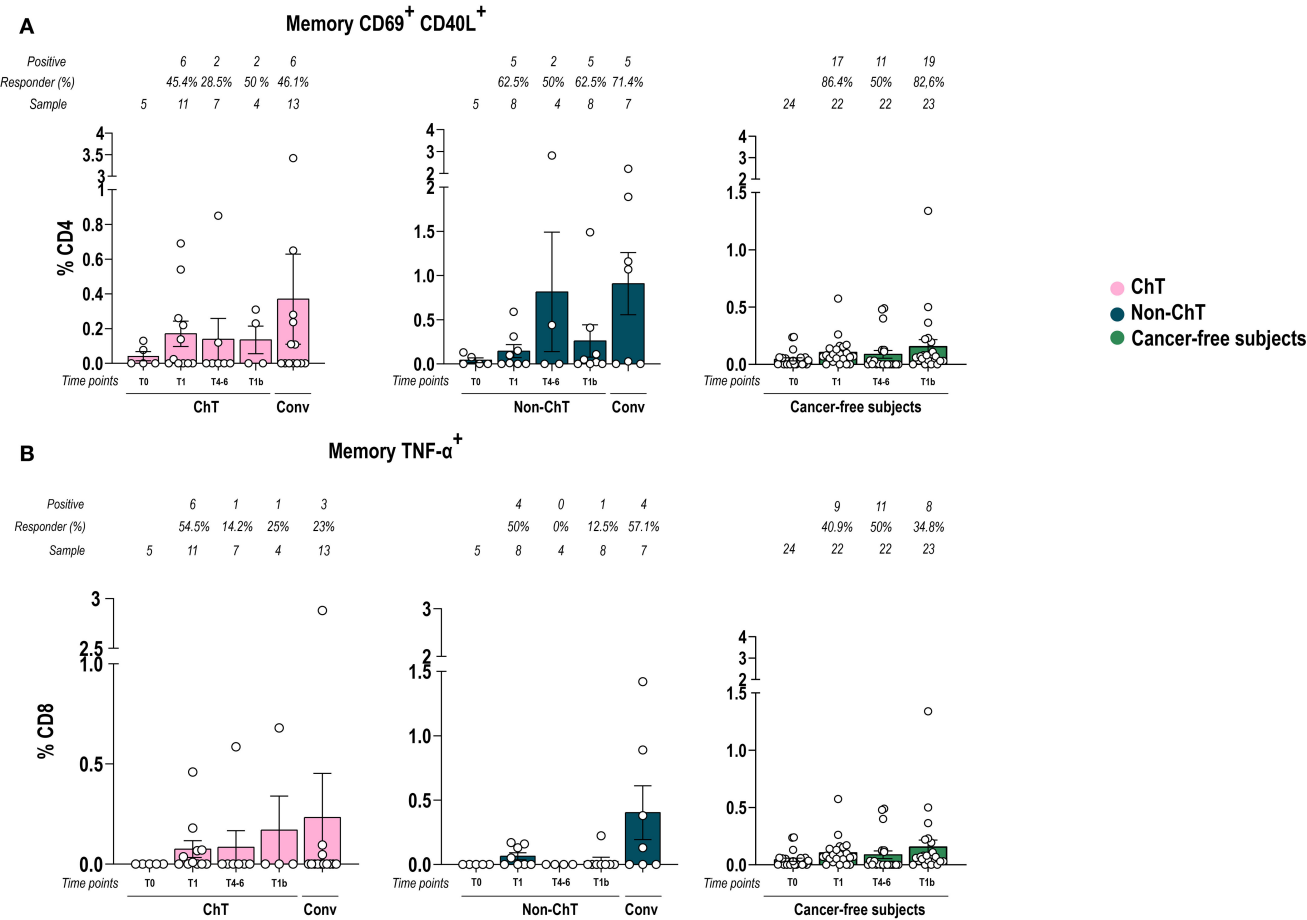
Vaccination with BNT162b2 mRNA induced a spike-specificity in T cells. Memory CD4^+^ AIM^+^ **(A)** and CD8^+^ memory TNF-α **(B)** spike-specific T cell frequencies over time in patients with cancer and in cancer-free subjects. Data are represented as scatter dot plots showing mean with SEM and individual values. One-way ANOVA statistical test was used to compare pre-and post-vaccination samples in each cohort at T0, T1, T4-6, T1b. Tukey’s correction was used for multiple comparisons testing. Significance levels are as follows: ****p <.0001, ***p <.001, **p <.01, * p <.05. Absence of significance symbols indicates that comparisons did not reach statistical significance.

Based on the absence of significant differences in the global T cell compartment, we then focused on measuring the frequency of spike-specific (S^+^) CD4^+^ and CD8^+^ T cells following stimulation with a peptide pool spanning the entire spike sequence. We analyzed the response at T1, T4–6 and T1b in ChT and non-ChT patients. The frequency of S^+^ CD4^+^ T cells is based on the expression of activation-induced markers (AIM) CD69 in combination with CD40L upon stimulation. In both oncologic groups, the frequencies of CD4^+^ AIM^+^ Memory S^+^ T cells fluctuated along time points following vaccination. At T4-6, one individual in the non-ChT group displayed an unusually high frequency, although this did not reflect a broader group-level trend (p > 0.05) (**Figure 4A**). In both cohorts, this frequency tended to be higher in convalescent patients rather than post-vaccination. Regarding CD8^+^ AIM^+^ S^+^ T cells, we measured those producing Tumor necrosis factor (TNF-α) (**Figure 4B**). In non-ChT patients, the frequency of CD8^+^ AIM^+^S^+^ T cells tended to increase at T1, and then did not further increase, while it fluctuated in ChT patients. Similarly to CD69 CD40L, levels of CD8 S^+^ Specific cells tended to be higher in convalescent patients, suggesting that natural infection elicited a sustained memory response.

### 3.5 *Immunophenotype and* sp*ike-*specificity of B cells elicited by vaccination

We then analysed the phenotype of B lymphocytes, as done for T cells. Multiparametric analysis showed that plasmablasts (CD19^+^ CD27^+^ CD38^+^ CD138^-^) decreased at T4-6, then tended to increase after the booster doses (T1b), bringing them back to the same percentages as the convalescents, while plasmacells (CD19^+^ CD27^+^ CD38^+^ CD138^+^) showed fluctuations with a more scattered distribution in ChT patients.

Memory B cells (CD19^+^ CD27^+^) decreased at T4–6 in ChT patients compared to non-ChT patients (p < 0.05). Total memory IgG cells tended to decrease over time in non-ChT patients, while in ChT patients such decrease was only observed at T4-6. Memory IgM remains unchanged in non-ChT patients and slightly decreases at T4–6 in ChT patients (**Supplementary Figure S7**).

**Figure 5:**
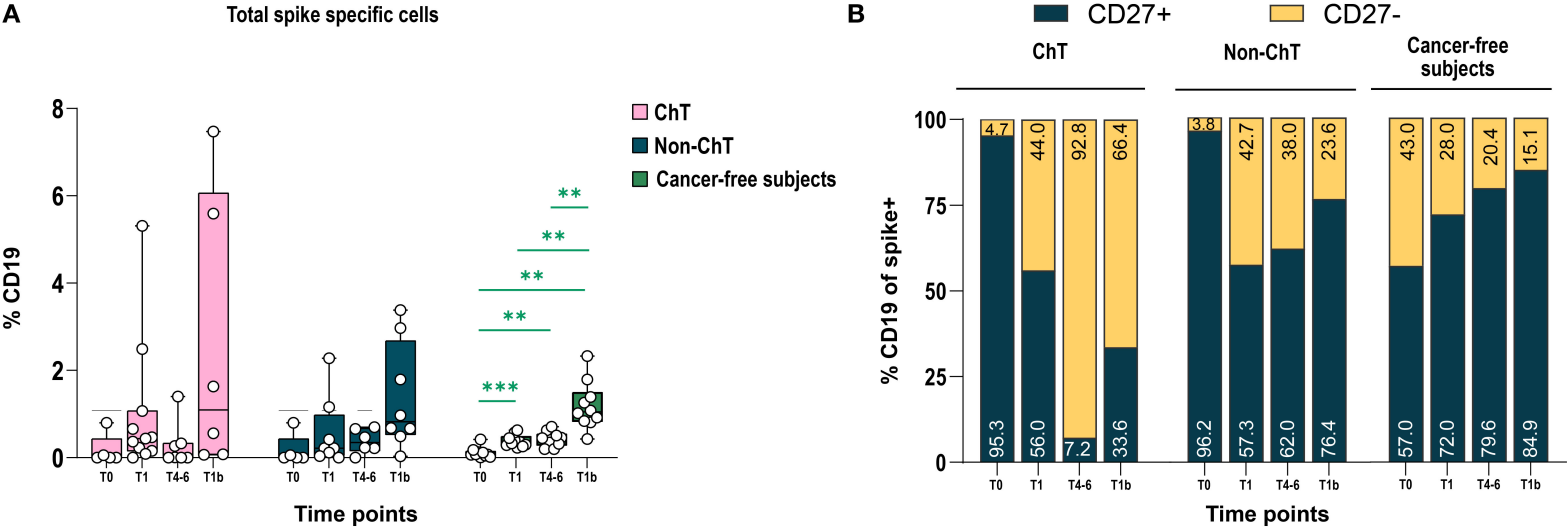
BNT162b2 mRNA vaccination elicited spike-specific B cells. **(A)** Percentage of Spike-specific cells in ChT, non-ChT patients, and cancer-free subjects. **(B)** Manual gating for the expression of CD27 in total spike specific cells. Time points are represented as T0, T1, T4–6 and T1b. Mixed-effects model was fitted to the data and used to compare pre-and post-vaccination samples in different cohorts (Fisher test). Multiple comparisons were corrected using Tukey’s tests. Significance levels are as follows: ****p <.0001, ***p <.001, **p <.01, *p <.05. Absence of significance symbols indicates that comparisons did not reach statistical significance.

We also investigated the phenotype of circulating spike-specific (S^+^) B lymphocytes elicited by vaccination in the entire B cell populations in ChT and non-ChT patients, using markers of activation and differentiation (CD27, CD21, CD38, CD138) and Ig class switch (IgD, IgM, IgG). The presence of S^+^ B lymphocytes was assessed by a simultaneous baiting of cells with recombinant spike, labelled with two different fluorochromes (either PE or PE-Cy7, respectively) to exclude a labelling-specific artefact. We observed that spike-specific B cells were induced at post-vaccination at T1 and further increased with the booster dose (T1b), with particularly high frequencies in some patients, although these differences did not reach statistical significance (**Figure 5A**). The decline of spike-specific B cells at T4–6 prompted us to investigate in detail the memory compartment (CD19^+^CD27^+^) of spike-specific B cells. We found a lower frequency of CD27^+^ cells on CD19 spike Specific cells in ChT individuals at T4–6 and T1b. In contrast the frequency of CD27^+^ B cells in non-ChT individuals was closer to that found in cancer-free controls (**Figure 5B**).

### 3.6 Unsupervised analysis of B cells

**Figure 6:**
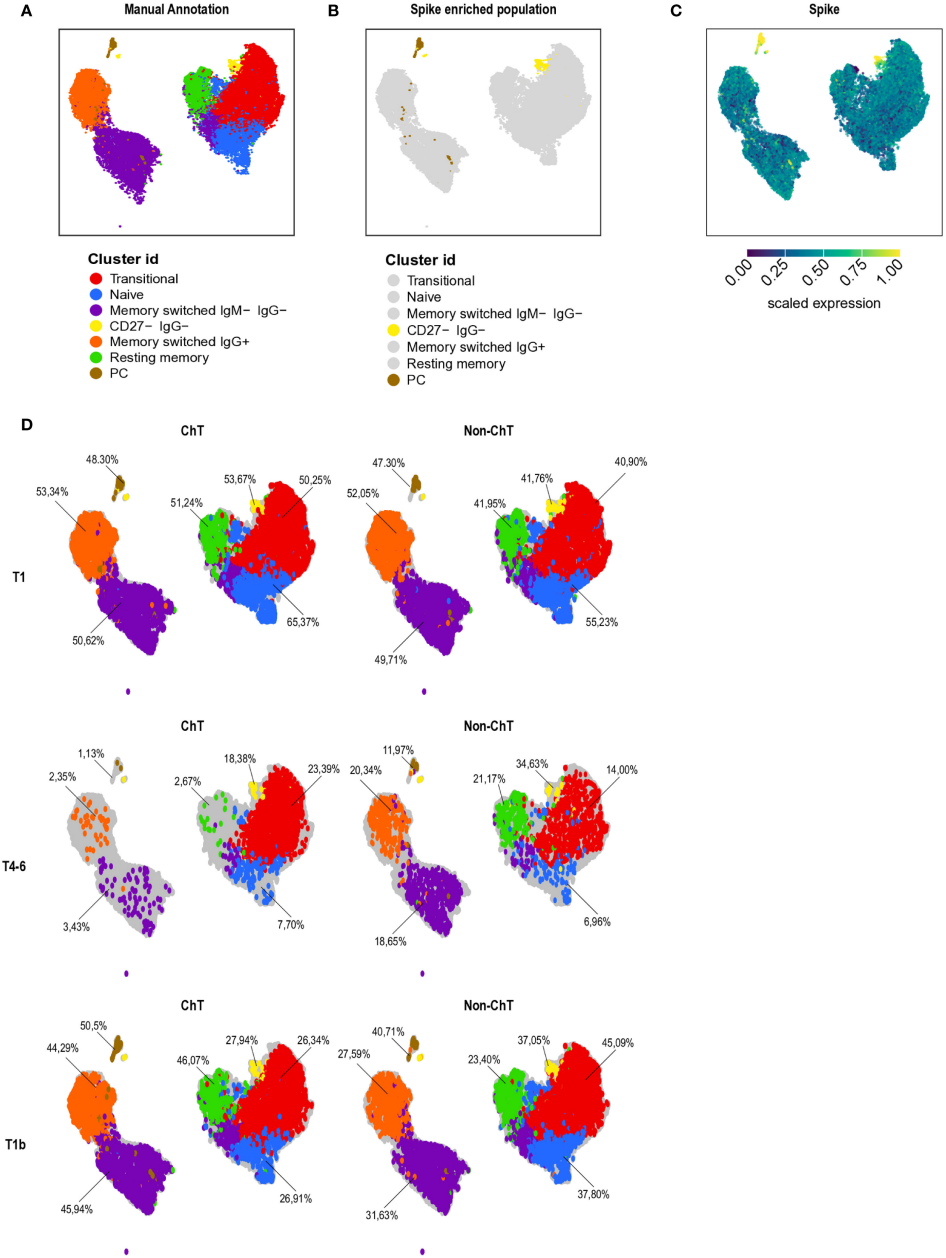
B cells composition in patients with cancer. Patients with cancer (N = 34, 17 ChT and 17 non-ChT samples) were analyzed at T1, T4–6 and T1b by multi-parametric flow cytometry and analyzed by Uniform Manifold Approximation and Projection (UMAP) to cluster cells based on cell positivity for canonical B-cell differentiation and maturation markers **(A)** UMAP map of all patients’ samples (1,038 cells, represented by each dot). In UMAP, the 7 identified B cell clusters are marked with different colors. **(B)** UMAP maps of spike-specific B cell colored in yellow and brown. **(C)** UMAP of spike specific expression. Yellow indicates high, and blue low expression levels. **(D)** UMAP of B cell cluster at different time point in ChT and non-ChT patients.

To gain a global and unbiased picture of the different B cell subset in entire data set, we conducted an unsupervised analysis of the flow cytometry data using dimensionality reduction (see Materials and Methods). By analyzing 17 ChT and 17 non-ChT samples, we identified distinct B lymphocyte clusters based on cell positivity to canonical B cell markers of differentiation and maturation markers. After manual annotation, we were able to identify 7 major sub-populations (**Figure 6A**, **Supplementary Figure S8**): Transitional CD27^-^ IgDCD38^hi^ CD21^+^, Naïve CD27^-^ IgD^+^ IgM^+^ CD38^lo^, Memory switched IgG- IgM- (CD27^+^ CD38^lo/-^ IgM^-^ IgG^-^), Memory switched IgG^+^, Resting memory CD27^+^ IgM^+^ CD21^+^, Plasmacells (PC) CD27^+^ CD38^+^ CD138^+^, and PC-like cells with low expression of CD27 (CD38^+^ CD138^+^ CD27^-^ IgG^-^, referred as CD27^-^ IgG^-^). Interestingly, spike-specific cells (considering S^+^ cells detected with both PE and PE-Cy7) mainly belonged to the PC and CD27^-^ IgG^-^ cluster (**Figures 6B, C**).

We then analyzed the expression pattern of the B cell clusters in oncologic groups along the vaccination period. There were strong differences in the frequencies of the plasmacell cluster and the memory compartment in ChT patients compared to non-ChT at T4-6 (**Figure 6D** **).** The most evident alteration was the expression level of CD27, a marker of activation and memory differentiation. We observed a higher expression of this marker in the memory compartment of non-ChT patients, at T4-6 (**Supplementary Figures S8**, **S9A, B**).

## 4 Discussion

In this study we comprehensively characterized the humoral and cellular immune response to the first three doses of the COVID-19 BNT162b2 vaccine in patients affected by solid cancer undergoing ChT or non-ChT treatments, as compared with a control group of cancer-free subjects. Our intent was to identify immunological determinants underlying differences in the immune response to vaccination.

Among the approved COVID-19 vaccines, the Pfizer/BioNTech (Comirnaty, BNT162b2) mRNA vaccine was one of the most widely used during the global vaccination campaign due to its efficacy and tolerability. Initially designed as a monovalent vaccine targeting the original Wuhan-Hu-1 (MN908947) spike protein of the SARS-CoV-2 virus, it was administered in two intramuscular doses to elicit neutralizing antibody and generate an effective B and T-cell response (28). However, waning immunity and the continued emergence of VoCs prompted the widespread adoption of a third booster dose of vaccine to enhance and prolong immunologic memory. This booster increased protection against Delta and Omicron variants, reducing disease severity in most cases. More recently, a fourth dose was recommended for vulnerable populations and healthcare workers, using either the monovalent or the newer bivalent formulation targeting both the ancestral strain and the Omicron VoC (29).

Patients with cancer are known to be more susceptible to severe COVID-19 and reinfection than cancer-free controls; thereby, they were given priority for COVID-19 vaccination in late 2020/early 2021. Owing to disease-associated and therapy-induced immune impairment, patients with cancer are less likely to mount a proficient immune response upon vaccination than the general population. In the light of the waning antibody responses and the inherently higher risk of suboptimal immunity in this population, patients with cancer have been globally prioritized for booster vaccination to achieve immunity levels similar to that observed in the general population. The recommendation of repeated administrations also applies to other vaccines, including influenza, pneumococcal infection, hepatitis B or zoster reactivation, where evidence demonstrates that additional doses benefited this population (30–34).

Data from initial studies in cancer patients receiving COVID-19 vaccines suggested that the decline in immune response over the months following vaccination showed a comparable dynamic to that observed in the general population (35), though with more pronounced waning (36). Moreover, vaccine-induced immune responses are commonly impaired by several cancer therapies, with ChT generally regarded as the most detrimental to the immune system compared to targeted therapies and immunotherapy (11, 37). Since a robust anti-S antibody response with neutralizing activity is an acknowledged correlate of protective immunity against SARS-CoV-2 infection, many studies of COVID-19 vaccination in cancer patients have focused on the antibody response induced against the ancestral SARS-CoV-2 strain and VoCs, including Omicron (18, 36, 38, 39). In these studies, cancer patients who received three vaccine doses showed lower antibody titers compared to cancer-free controls who were given the same regimen. Neutralizing antibodies were clearly detected in cancer patients, with titers declining over time alongside susceptibility to arising VoC, as for cancer-free controls.

In line with other groups, our data show that patients with cancer develop anti-S and anti-RBD (Receptor Binding Domain) antibodies, with levels declining four-six months after primary vaccination, and rising again after the booster dose, with a dynamic similar to cancer-free subjects (40, 41). In these patients, antibody response levels were higher than those observed in convalescent patients, independently of the treatment type (ChT vs non-ChT).

Interestingly, while the capability to elicit antibodies recognizing full-length spike Wuhan, Delta and Omicron VoC was comparable between cancer patients and cancer-free subjects, we found that the anti-RBD response was deficient in the former (42). Such difference in the level of anti-RBD antibodies was particularly pronounced in ChT-treated patients compared to those receiving non-ChT agents. Since the RBD binds the ACE2 receptor on human cells to allow viral entry, there is a clear correlation between levels of anti-RBD antibodies, neutralization activity, and cross protection against SARS-CoV-2 VoC (43). This suggests that a lower antibody response to this protein region is likely to result in a less effective protection against the virus. To address this point, we investigated the serum neutralization activity four to six months after completing the primary vaccination series (two doses) and determined the potency index. It is well established that the humoral response undergoes continuous development and maturation long after initial antigen exposure, with memory B cells showing improved quality and breadth at later compared to earlier time points (44, 45). In our analysis, we observed a higher potency index for the Wuhan lineage (ND50/spike IgG and ND50/RBD IgG) in non-ChT patients compared with ChT patients. This indicates that a greater proportion of spike- and RBD-specific antibodies are also neutralizing in patients not receiving ChT, likely reflecting a more efficient development of memory B cells, which may be impaired by ChT. In contrast, no difference was observed in the serum neutralizing activity between the two cohorts against omicron VoCs, likely due to the prevailing antibody-binding escape mechanisms driven by mutations in the RBD region.

Concerning the T cell response elicited by COVID-19 vaccination, its critical role in the durability and recall of memory response to reinfection is widely acknowledged (46). Reports in the general population have shown that T cell epitopes are more broadly conserved (47) and less overtly affected by VoC compared to B cell epitopes.

In cancer patients, a limited number of studies investigated in depth the cellular immune response, sometimes reporting discordant antibody and cellular responses (18). These studies investigated the release of effector cytokines, such as IFNγ, alone or combined to IL-2a and TNFα release. A recent study reported that patients with cancer showed a reduced frequency of multifunctional T cells producing multiple cytokines, with a predominance of monofunctional T cells producing TNFα over multifunctional cells producing different effector cytokines (48). The T-cell response was also measured in patients with cancer based on activation markers of immune cells, revealing that a significant fraction (46–79%) of patients with solid tumors elicited a detectable T-cell response (49). Notably, this response was consistently more robust than the antibody response, since 30–75% of seronegative patients had measurable specific T-cell responses to vaccination, independent of disease subtype (50–52). Detectable T cell levels were reported in the absence of antibody responses in patients with cancer, although with lower frequency in those receiving ChT than immune checkpoint inhibitors (39). In line with these findings, our study detected spike-specific memory CD4^+^ and CD8^+^ T cells, which, although present at low frequencies, expressed the activation markers CD69 and CD40L or produced TNF-α in a lower proportion of the ChT group compared to the non-ChT group. As a trend, these cells were found more commonly and at higher frequencies in vaccinated individual rather than in convalescents, suggesting distinct dynamics between vaccination and natural viral infection.

Compared to other studies, our research focused on the phenotype of spike-specific effector B-cells, which play a crucial role in mediating protection. On average, spike-specific CD19^+^ cells showed a comparable profile in patients with cancer vs cancer-free patients, highlighting the beneficial effect of the booster dose. By using an unbiased bioinformatic analysis of flow cytometry data combined to manual annotation, we identified seven major spike-specific subpopulations, including Transitional, Naïve, Memory switched IgG- IgM-, Memory switched IgG^+^, CD27^-^ IgG^-^, Resting memory, and Plasmacells (PC). The PC subpopulation was the most represented among spike-specific CD19^+^ cells, as expected. Interestingly, by performing this analysis we identify a subset of spike-specific B cells clustering with plasma cells but negative for the CD27 marker, whose role is not clear. In support of our finding, Zurbuchen Y. et al. (53) recently described that the B cell response to SARS-CoV-2 and COVID-19 vaccines, and to other pathogens as well, may adopt tailored effector mechanisms, resulting in functionally specialized memory B cell subsets. Notably, CD21^+/–^ CD27^–^ memory B cells were shown to originate from spike-specific CD21^+^/CD27^+^ memory B cells, which can redifferentiate into other memory subsets upon antigen rechallenge, demonstrating that single memory B cell clones can follow distinct functional trajectories over time.

As a general remark spike^+^ B cells and PC cells displayed a lower expression level of CD27 in the chemotherapy group. CD27, a member of the TNF receptor superfamily (TNFRSF), is a Type 1 transmembrane glycoprotein that interacts with CD70 and generally promotes maturation and activation of T and B lymphocytes (54, 55). The CD27-CD70 interaction is crucial as a co-stimulatory signal for both B-cell and T-cell activation (56). In B cells, a higher production of both CD27 and CD70 results in higher production of immunoglobulins, which is presumably essential for the activation of B cells and the formation of memory B cells. Deficiencies of CD27 and CD70 have been shown to cause severe consequences for the memory B cell compartment (57). Based on the crucial role of CD27, we may hypothesize that patients receiving cytotoxic ChT elicit a less robust activation and differentiation to a memory state, while this phenomenon is not observed when patients with cancer are treated with chemo-free regimens. Additional investigations are needed to understand whether this ChT-linked impaired response diminishes the capability to confer long-term immunity against SARS-CoV-2 reinfection, possibly also limiting the ability to sustain a durable neutralizing antibody response.

The primary limitation of this study is the relatively small number of patient samples undergoing detailed characterization of immune responses. Such limited sample size prevented more accurate stratification based on treatment regimens and other clinical features. Furthermore, the higher age of cancer patients compared to non-cancer controls could have contributed to subtle differences in immune baseline and should be considered when interpreting the findings. Additionally, the study design did not consistently allow for longitudinal sampling from the same patients, which may have affected the evaluation of immune dynamics over time. However, for patients with longitudinal samples, treatment group (ChT vs non-ChT) was assigned dynamically at each time point based on actual treatment exposure to account for potential treatment changes over time (e.g., line switch due to progression), thereby minimizing misclassification and ensuring accurate interpretation of immune responses. As an additional limitation, the timepoints chosen for sample collection were based on prior evidence from our group and others (11–14) regarding the kinetics of vaccine-induced immune responses; however, we acknowledge that these intervals remain somewhat arbitrary. While we believe they adequately capture key phases of the immune trajectory, a denser sampling strategy might have provided additional resolution, particularly regarding transient or heterogeneous immune features. Finally, the observed alterations in antibody titers and CD27 expression raise biologically meaningful questions that warrant future investigation through targeted studies in cellular systems, animal models, or longitudinal patient cohorts. Despite these limitations, our study offers valuable immunological insights into a less robust immune response to COVID-19 vaccination in patients with solid tumors undergoing cancer therapies, particularly those receiving ChT. Furthermore, it underscores the importance of regular booster doses to maintain protection against SARS-CoV-2 infection and severe COVID-19 disease in this vulnerable high-risk population.

## Data Availability

The raw data supporting the conclusions of this article will be made available by the authors, without undue reservation.
