# Supplementary material for: Impact of chemotherapy on humoral and cellular immune responses to COVID-19 vaccination in patients with solid tumors
Source: Front Immunol. 2025 Sep 25;16:1664072. doi: 10.3389/fimmu.2025.1664072 (PMC12507793; doi:10.3389/fimmu.2025.1664072)
Supplement: Supplementary file 1 [file DataSheet1.docx]

**Supplementary Materials**

**Impact of Chemotherapy on Humoral and Cellular Immune Responses to COVID-19 Vaccination in Patients with Solid Tumors**

Andrea Favalli^1^*, Giorgio Patelli^2,3^*, Gruarin Paola^1^, Andrea Gobbini^1^, Elisa Pesce^4^, Sara Mariano^3^, Mauro Bombaci^1^, Francesca Vincenti^1^, Lorena Donnici^1^, Silvia Marchese^1,5^, Daniele Piscazzi^2,3^, Alessio Amatu^3^, Federica Tosi^3^, Silvia Ghezzi^3^, Arianna Pani^3^, Silvia Principato^1^, Andrea Lombardi^6,7^, Alessandra Bandera^6,7^, Sergio Abrignani^1,4^°, Salvatore Siena^2,3^°, Andrea Sartore-Bianchi^2,3^°^ and Renata Grifantini^1,8^°^.

**Affiliations:**

^1^INGM, Istituto Nazionale Genetica Molecolare "Romeo ed Enrica Invernizzi", Milan, Italy.

^2^Department of Oncology and Hemato-Oncology, Università degli Studi di Milano, Milan, Italy.

^3^⁠Niguarda Cancer Center, Grande Ospedale Metropolitano Niguarda, Milan, Italy.

^4^Department of Clinical Sciences and Community Health, University of Milan, Milan, Italy.

^5^ DiSFeB Dipartimento di scienze farmacologiche e biomolecolari, Università degli studi di Milano, Milan, Italy.

^6^Infectious Diseases Unit, Foundation IRCCS Ca' Granda Ospedale Maggiore Policlinico, Milan, Italy.

^7^Department of Pathophysiology and Transplantation, University of Milan, Milan, Italy.

^8^CheckmAb Srl, Milan, Italy.

*equally contributed as first authors.

°equally contributed as senior authors.

^co-corresponding authors.

This file includes:

- Extended Materials and Methods
- *Sample collection, processing, and storage*
- *Determination of anti-spike and anti-RBD IgG titers by* *Enzyme-linked immunosorbent assay (ELISA)*
- *SARS-CoV-2 pseudovirus neutralization assay*
- *Immunophenotyping by high-dimensional flow cytometry*
- *Analysis of spike-specific T cell response*
- **Table S1**
- **Figures** **S1 to S9**

**Extended Materials and Method**

*Sample collection, processing, and storage*

Peripheral blood samples were collected from all participants. Approximately 50 mL of peripheral blood were drawn into ethylenediaminetetraacetic acid (EDTA)-treated 10 mL tubes to prevent coagulation. Additional 10 mL of blood were drawn in a vacutainer tube without anticoagulant to separate serum. In any case, blood sampling was performed before antineoplastic treatment administration, if the latter was planned for the same day. All samples were pseudo-anonymized to maintain patient confidentiality, following relevant ethical statements that are already described in master protocols.

Upon collection, plasma and peripheral blood mononuclear cells (PBMCs) were isolated using centrifugation at 2000 rpm for 20 minutes at room temperature, and Ficoll-Paque density gradient medium. PBMCs were counted, viability checked and cryopreserved in a cryoprotective medium in liquid nitrogen for later use. Serum separator tubes were centrifuged, the supernatant was collected and stored at -80°C for subsequent analyses.

*Determination of anti-spike and anti-RBD IgG titers by* *Enzyme-linked immunosorbent assay (ELISA)*

Anti-S and anti-RBD immunoglobulin G (IgG) sera titers were determined by ELISA for Wuhan (MN908947, Wuhan-Hu-1), Delta and Omicron (alias B.1.1.529) SARS-CoV-2 variants. The whole procedure was automatically performed with a Freedom-EVO Liquid Handling system (Tecan). In brief, 96-well plates were coated with 100μl/well purified recombinant protein solution (2.5μg/ml in phosphate-buffered saline (PBS)), and incubated overnight at 4°C. After blocking with PBS-5% bovine serum albumin (BSA), sera were serially diluted and incubated for 1h at 37°C. Eight serial 2-fold dilutions starting from 1:100 were tested for each sample in duplicate. Plates were washed with PBS/Tween 0.05% and probed with horseradish peroxidase–conjugated anti-human IgG secondary antibody (1:1000 in PBS/BSA 1%/Tween 0.05%) for 40 min RT. After washing, the reaction was developed with 100μl of 3,3′,5,5′-tetramethylbenzidine (TMB, ThermoFisher) for 10 min, stopped with 100μl of 1M H2SO4, and the absorbance was measured at λ = 450 nm by Infinite F200 PRO instrument (Tecan). Absorbance values higher than the mean + 2 standard deviation (SD) of negative controls were considered as positive and used as Limit of Quantification (LoQ).

*SARS-CoV-2 pseudovirus neutralization assay*

For SARS-CoV-2 pseudovirus generation HEK-293TN cells were co-transfected with 32 µg of reporter plasmid pLenti CMV-GFP-TAV2A-LUC-Hygro, 12.5 µg of pMDLg/pRRE (Addgene #12251), 6.25 µg of pRSV-Rev (Addgene #12253) and 9 μg pcDNA3.1_spike_del19 (Addgene #155297) in a cell culture dish. Supernatant was collected 30h after transfection. Pseudovirus (BA.5) preparation was titrated by infecting HEK293TN-hACE2 cells with 3-fold serial dilutions and measuring the number of GFP-positive cells by FACS analysis. Briefly, dilutions of serum (starting from 1:4 Use as Limit of quantification of serum neutralizing antibodies) were incubated with SARS-CoV-2 pseudovirus 1h at 37°C and transferred to HEK293TN-hACE23 pre-plated in a white 96-well plate. Cells were infected at MOI 0.1. Luminescence was measured 24h after infection by Bright-Glo™ Luciferase Assay System (Promega). Obtained RLUs were normalized to not treated (PBS) controls and dose response curves were generated by nonlinear regression curve fitting to calculate ND50. The titers of neutralizing sera ere expressed as neutralization dilutions reducing infection by 50% (ND50).

*Immunophenotyping by high-dimensional flow cytometry*

Frozen PBMCs were thawed, washed in Fluorescence-Activated Cell Sorting (FACS) buffer and incubated with Fixable Viability Stain 780 (BD Horizon, cat. no. 565388) diluted 1:2000 in PBS at room temperature in the dark. After 15 min, cells were washed in PBS. For surface marker detection, cells were incubated in Brilliant Stain Buffer (BD Horizon, cat. no. 566349) diluted 1:2 in PBS supplemented with antibodies for 30 min at room temperature in the dark. Cells were then washed in PBS and fixed 15 min at 4°C using eBioscience FOXP3 staining kit according to the manufacturer protocol (eBioscience, cat. no. 00-5523). To detect intracellular factors (GZMK, GZMB, T-BET) a further incubation in Permeabilization Reagent (eBioscience, cat. no. 00-833) supplemented with antibodies was performed for 30 min at 4°C. Samples were acquired on a BD FACSymphony A5 flow cytometer (BD Biosciences) equipped with 5 lasers (UV, 350 nm; violet, 405 nm; blue, 488; yellow/green, 561 nm; red, 640 nm). Antibodies used for high-dimensional flow cytometry analyses are listed in **Table S1**. Gating strategies for flow cytometry analysis of T and B cells are shown in **Figure S1, S2** and **S3.**

*Analysis of spike-specific T cell response*

To evaluate the expression of activation-induced markers (AIM), PBMCs resuspended in RPMI complete medium (2 mM glutamine, 1 mM sodium pyruvate, 1% non-essential amino acids, 1% penicillin/streptomycin) supplemented with 10% of FBS (Fetal Bovine Serum) were plated at 1x106 cells/well into 96 well round bottom and stimulated with SARS-CoV-2 peptide pools (2 µg/ml peptide Miltenyi Biotec, cat. no. 130-126-701). The medium containing dimethyl sulfoxide (DMSO) or Staphylococcal Enterotoxin B (SEB, 1 µg/ml Sigma Aldrich, cat. no. S4881) was used as negative and positive control, respectively. Brefeldin A (BFA, Sigma Aldrich, cat. no. B7651) was added to cultures (final concentration 10 µg/ml) after 2h of stimulation and cells were incubated o/n at 37°C. After incubation cells were harvested and stained with Fixable Viability Stain 780 (BD Horizon, cat. no.565388) diluted 1:2000 in PBS at room temperature in the dark. For surface marker detection, cells were incubated with an antibody cocktail containing CD3 BUV805, CD8 BUV563, CD4 BUV737, CD69 BV650, CCR7 BV711 and CD45RA BUV496. After cell washing and fixing, intracellular staining for CD40L PE-Cy5, TNF-a BUV-395 was performed. Samples were analyzed on a BD FACSymphony A5 flow cytometer (BD Biosciences). Data are presented as (% cells in peptide stimulated samples) – (% cells in DMSO treated samples).

The LoQ for antigen-specific CD4+/CD8+ T and B cell responses (%) was calculated using the mean plus one standard deviation in pre-vaccine unexposed donors, corresponding to 0,0001%. The gating strategy for the dentification of spike-specific T cells is represented in **Figure S3**.

**Table S1 – List of antibodies used for immunophenotypic analyses.**

| **Antibody target**  **(eventual alias in brackets)** | **Fluorochrome** | **Clone** | **Company** | **Catalog #** |
| --- | --- | --- | --- | --- |
| **CD127** | BB700 | HIL-7R-M21 | BD | 566398 |
| **CD137 (41BB)** | BUV615 | 4B4-1 LIGAND | BD | 751492 |
| **CD183 (CXCR3)** | PECY7 | 1C6/CXCR3 | BD | 560831 |
| **CD185 (CXCR5)** | APC-CY7 | J252D4 | BioLegend | 356926 |
| **CD19** | APC-Vio 770 | LT19 | Miltenyi | 130-128-022 |
| **CD195 (CCR5)** | FITC | HEK/1/85a | BioLegend | 313705 |
| **CD196 (CCR6)** | APCR700 | 11A9 | BD | 565173 |
| **CD197 (CCR7)** | BV711 | 150503 | BD | 566602 |
| **CD21** | PECY5 | B-ly4 | BD | 551064 |
| **CD138** | Pe-CF594 | MI15 | BD | 564606 |
| **CD25** | Pecy5 | M-A251 | BD | 555433 |
| **CD27** | BUV737 | L128 | BD | 564301 |
| **CD27** | Vio Bright  FITC | M-T271 | Miltenyi | 130-128-022 |
| **CD279 (PD1)** | BV650 | EH12.1 | BD | 564104 |
| **CD3** | BUV 805 | UCHT1 | BD | 612895 |
| **CD4** | BUV 395 | SK3 | BD | 563550 |
| **CD4** | BUV737 | SK3 | BD | 612748 |
| **CD40L** | PECY5 | cl.24-31 | BioLegend | 310808 |
| **CD45RA** | BUV496 | HI100 | BD | 750258 |
| **CD45RO** | BV605 | UCHL1 | BD | 562791 |
| **CD69** | BV650 | FN50 | BD | 563835 |
| **CD8** | BUV 563 | RPA-T8 | BD | 612914 |
| **FOXP3** | PE-CF594 | 259D/C7 | BD | 562421 |
| **GRANZYME B** | BV421 | GB 11 | BD | 563389 |
| **GRANZYME K** | Alexa Fluor647 | G3H69 | BD | 566655 |
| **IFN-γ** | PE | 4S.B3 | BioLegend | 502509 |
| **IgD** | BV480 | IA6-2 | BD | 566138 |
| **IgG** | VioBlue | IS11-3B2.2.3 | Miltenyi | 130-128-022 |
| **IgM** | APC | PJ2-22H3 | Miltenyi | 130-128-022 |
| **LIVE AND DEAD** | FIX VIAB 780 | - | BD | 565388 |
| **LIVE AND DEAD** | Aqua | - | thermofisher | L34957 |
| **TNF-α** | BUV395 | MAb11 | BD | 563996 |

**Figure S1.** Gating strategies for flow cytometry analyses used for the identification of total spike-specific B lymphocytes and spike-specific memory B cell based on CD27 expression.

**Figure S2.** Gating strategies for flow cytometry analyses used for the identification of: A) CD4^+^ T cell TH1, TH1*and TH17 B) TREG C) Tfh Like and CXCR5 subsets D) CD4^+^ T cell CCR5^+^ and CXCR3^+^ CCR5^+^; E) CD8^+^ Total Memory GZMK^+^ and GZMB^+^.

**Figure S3.** Gating strategies for flow cytometry analyses of S-specific T cell response. A) Identification of Memory CD4^+^ and B) Memory CD8^+^ AIM^+^ S^+^ T cells.

**Figure S4.** Schematic overview of vaccination schedule and study design.

Patients with cancer and healthy donors vaccinated individuals received two doses of the BNT162b2 vaccine, administered 21 days apart, and a booster at least 6-8 months after the first dose. Blood samples were collected before vaccination (T0), one-two months after primary vaccination (T1), four-six months after primary vaccination (T4-6), and 1 month after the booster dose (T1b). For comparison, blood samples were also collected from SARS-CoV-2 convalescent subjects with or without cancer (20-50 days from a positive swab). Serological responses to vaccination were measured by serum antibody titration and neutralization assay. PBMCs were used for immunophenotype and for antigen specific analyses of T and B cells.

**Figure S5:** Phenotypic analysis of CD4^+^T cells identifying immune signatures in SARS-CoV-2 patients with cancer and cancer-free vaccinated patients.

Relative abundance of A) Th1 (CCR6^-^ CXCR3^+^), B) Th17 (CCR6^+^ CXCR3^-^), C) Th1*(CCR6^+^ CXCR3^+^), D) Treg (CD127^-^, CD25^+^), E) Tfh Like (CXCR5^+^), and F) CD4 CCR5^+^ subpopulations are reported as % of CD4. Data are represented as box and whiskers showing median, min to max and individual values. Mixed-effect analysis was used to compare unpaired samples between ChT, non-ChT and cancer-free groups. Multiple intragroup comparisons were corrected using Tukey’s tests. Green, pink and blue lines indicate statistical comparisons between time points in cancer-free, ChT and non-ChT groups. Each dot represents a single individual. Comparisons between different groups at the same time point are indicated by the colour-coded asterisk. Significance levels are as follows: ****p < .0001, ***p < .001, **p < .01, *p < .05. Blue asterisk indicates statistical difference between time points in the non-ChT group; pink asterisk indicates statistical difference between time points in the ChT group; green asterisk indicates statistical difference between time points in the cancer-free group.

Major observations: We found that the frequency of the Th1 effector subset, which is essential for defense against viral infections, fluctuated during vaccination in both ChT and non-ChT groups, with an increasing trend at T1 (post-primary vaccination) and a decrease at T4-6, while in cancer-free subjects Th1 progressively increased over time from T1 to T1b. Additionally, in patients with cancer, post-vaccination Th1 levels failed to exceed those of convalescent subjects, in contrast to cancer-free individuals, where a significant increase was observed **(Panel A)**. Th17 cells, which are associated with enhanced vaccine efficacy (27), progressively increased over time post-vaccination, regardless of ChT or non-ChT treatment. Such increase was heterogeneous, but generally higher compared to cancer-free controls **(Panel B)**. The frequencies of Th1* cells post-primary vaccination showed a sustained profile up to T4-6 and then dropped at T1b in all groups **(Panel C)**.

**Figure S6.** Phenotypic analysis of CD8^+^ T cells identify immune signatures in SARS-CoV-2 patients with cancer and cancer-free vaccinated subjects.

Relative abundance of A) CD8^+^ effectors expressing GzmB and B) CD8^+^ effector memory expressing GzmK is reported as % of CD8 Total memory. Data are represented as box and whiskers showing median, min to max and individual values. Mixed-effect analysis was used to compare unpaired samples between ChT, non-ChT and cancer-free groups. Multiple intragroup comparisons were corrected using Tukey’s tests. Green, pink and blue lines indicate statistical comparisons between time points in cancer-free, ChT and non-ChT groups. Each dot represents a single individual. Comparisons between different groups at the same time point are indicated by the colour-coded asterisk. Significance levels are as follows: ****p < .0001, ***p < .001, **p < .01, *p < .05. Blue asterisk indicates statistical difference between time points in the non-ChT group; pink asterisk indicates statistical difference between time points in the ChT group; green asterisk indicates statistical difference between time points in the cancer-free group.

Major observation: We noticed a fluctuation in CD8 effector memory cells expressing Granzyme B post-vaccination in both oncologic groups, which was less evident in the cancer-free subjects. We also noticed a trend towards increased percentage of CD8 effector memory cells expressing Granzyme K at T1 (after primary vaccination) in patients with cancer, particularly in the non-ChT ones, while this cell population showed a sustained growth post-vaccination in cancer-free subjects.

**Figure S7.** Phenotypic analysis of B cells in patients with cancer.

A) Plasmablasts (CD19^+^ CD20^-^ CD27^+^ CD38^+^ CD138^-^), B) Plasmacells (CD19^+^ CD20^-^ CD27^+^ CD38^+^ CD138^+^), C) Memory B cells (CD19^+^ CD27^+^), D) Memory IgG (CD19^+^ CD27^+^ IgG^+^) and D) Memory IgM (CD19^+^ CD27^+^ IgM^+^); all reported as percentage of CD19. Data are represented as box and whiskers showing median, min to max and individual values. Mixed-Effect analysis was used to compare pre-and post-vaccination samples in each cohort at T0, T1, T4-6, T1b. Tukey’s correction was used for multiple comparisons testing. Significance levels are as follows: ****p < .0001, ***p < .001, **p < .01, * p < .05.

**Figure S8.** Unbiased bioinformatic analysis of multiparametric flow cytometry data from B-cells of ChT (A) and non-ChT (B) patients (all n=34).

Spectrogram showing the expression levels of the 10 differentiation markers in 7 B-cell cluster.

**Figure S9.** A) Spectrogram on CD27 expression in different clusters and in ChT vs non-ChT patients. B) Boxplot of Memory compartment (Memory switched IgM^-^ IgG^-^, Memory switched IgG^+^ and resting memory) for the expression of CD27.
